# Supplementary material for: A two-tiered latent class and spatial analytical approach to identify clusters of neonatal mortality among very low birth weight infants: A population-based cohort study
Source: PLoS One. 2026 Feb 24;21(2):e0343004. doi: 10.1371/journal.pone.0343004 (PMC12931791; doi:10.1371/journal.pone.0343004)
Supplement: S1 Table — (DOCX) [file pone.0343004.s001.docx]

**S1 Table. ICD-10-Coded Diagnoses from Death Certificates and their Frequency in the Study Population.**

| **Category** | | **Subcategory** | **Description** | **Frequency** |
| --- | --- | --- | --- | --- |
| **INFECTION** | | | | |
| **A32** | | A32.7 | Listerial sepsis | 0 |
| **A40** | | A40.0-A40.9 | Streptoccoccal sepsis | 0 |
| **A41** | | A41.1-A41.9 | Other sepsis (Staphylococcus, *Haemophilus influenzae*, anaerobes, other Gram-negative organisms, other and unspecified) | 107 |
| **B37** | | B37.7 | Candidal sepsis | 3 |
| **G00** | | G00.0-G00.9 | Bacterial meningitis, not elsewhere classified | 16 |
| **G01** | |  | Meningitis in bacterial diseases classified elsewhere | 0 |
| **G04** | | G04.0-G04.9 | Encephalitis, myelitis and encephalomyelitis | 0 |
| **J13** | |  | Pneumonia due to *Streptococcus pneumoniae* | 0 |
| **J14** | |  | Pneumonia due to *Haemophilus influenzae* | 0 |
| **J15** | | J15.0-J15.9 | Bacterial pneumonia, not elsewhere classified | 3 |
| **J16** | | J16.0-J16.8 | Pneumonia due to other infectious organisms, not elsewhere classified | 1 |
| **J17** | | J17.0-J17.8 | Pneumonia in diseases classified elsewhere | 0 |
| **J18** | | J18.0-J18.9 | Pneumonia, organism unspecified | 307 |
| **M86** | | M86.0-M86.9 | Osteomyelitis | 0 |
| **N30** | | N30.0-N30.9 | Cystitis | 0 |
| **N39** | | N39.0 | Urinary tract infection, site not specified | 1 |
| **P23** | | P23.0-P23.9 | Congenital pneumonia | 577 |
| **P36** | | P36.0-P36.9 | Bacterial sepsis of newborn (Streptococcus, Staphylococcus, *Escherichia coli*, anaerobes, other and unspecified) | 14,888 |
| **P37** | | P37.2 | Neonatal disseminated listeriosis | 0 |
| **P38** | |  | Omphalitis of newborn with or without mild hemorrhage | 9 |
| **P39** | | P39.0-P39.9 | Other infectious specific to the perinatal period | 2,657 |
| INTRAPARTUM EVENTS | | | | |
| **P10** | P10.0-P10.9 | | Intracranial laceration and hemorrhage due to birth injury | 9 |
| **P11** | P11.0-P11.9 | | Other birth injuries to central nervous system | 2 |
| **P12** | P12.0-P12.9 | | Birth injury to scalp | 1 |
| **P13** | P13.0-P13.9 | | Birth injury to skeleton | 1 |
| **P14** | P14.0-P19.9 | | Birth injury to peripheral nervous system | 0 |
| **P15** | P15.0-P15.9 | | Other birth injuries | 284 |
| **P20** | P20.0-P20.9 | | Intrauterine hypoxia | 577 |
| **P21** | P21.0-P21.9 | | Birth asphyxia | 5,247 |
| **P24** | P24.0 | | Neonatal aspiration of meconium | 27 |
| MALFORMATIONS | | | | |
| **Q00-Q07** |  | | Congenital malformations of the nervous system | 879 |
| **Q10-Q18** |  | | Congenital malformation of eye, ear, face and neck | 45 |
| **Q20-Q28** |  | | Congenital malformation of the circulatory system | 1,650 |
| **Q30-Q34** |  | | Congenital malformation of the respiratory system | 535 |
| **Q35-Q37** |  | | Cleft lip and cleft palate | 51 |
| **Q38-Q45** |  | | Other congenital malformations of the digestive system | 327 |
| **Q50-Q56** |  | | Congenital malformations of genital organs | 41 |
| **Q60-Q64** |  | | Congenital malformations of the urinary system | 386 |
| **Q65-Q79** |  | | Congenital malformations and deformations of the musculoskeletal system | 558 |
| **Q80-Q89** |  | | Other congenital malformations | 1,469 |
| **Q90-Q99** |  | | Chromosomal abnormalities, not elsewhere classified | 347 |
| **RESPIRATORY FAILURE OR DISEASES** | | | | |
| **J43** | J43.0-J43.9 | | Emphysema | 0 |
| **J81** |  | | Pulmonary oedema | 6 |
| **J93** | J93.0-J93.9 | | Pneumothorax | 7 |
| **J96** | J96.0-J96.9 | | Respiratory failure, not elsewhere classified | 31 |
| **P07** | P07.2 | | Extreme immaturity | 11,076 |
| **P22** | P22.0-P22.9 | | Respiratory distress of newborn | 13,410 |
| **P25** | P25.0-P25.9 | | Interstitial emphysema and related conditions originating in the perinatal period | 1,896 |
| **P27** | P27.0-P27.9 | | Chronic respiratory disease originating in the perinatal period | 87 |
| **P28** | P28.0-P28.9 | | Other respiratory conditions originating in the perinatal period | 11,100 |
