# Supplementary material for: A two-tiered latent class and spatial analytical approach to identify clusters of neonatal mortality among very low birth weight infants: A population-based cohort study
Source: PLoS One. 2026 Feb 24;21(2):e0343004. doi: 10.1371/journal.pone.0343004 (PMC12931791; doi:10.1371/journal.pone.0343004)
Supplement: Attachment — Submitted filename: REVISION - PLOS ONE .pdf [file pone.0343004.s002.pdf]

I commend the authors on a well-written and thoughtfully structured manuscript. The study employs a sophisticated methodology and presents findings with significant utility for public health and the field of perinatology.
During my review, I identified several areas with potential for enhancement, which I outline below.

1. Adherence to the STROBE Statement
To ensure comprehensive reporting and identification of best practices, I recommend the authors explicitly confirm that all items of the STROBE checklist have been addressed.
Item 4 (Title and Abstract): The manuscript's title should clearly state the study design. While the use of Latent Class Analysis implies an observational design, the specific type (which I understand to be a retrospective cohort study) should be explicitly stated in the title, or if not possible, the abstract. This is a key requirement for immediate reader comprehension.
Other Items: Certain other STROBE criteria appear to be only partially met. These include a more detailed description of the sampling method and a clearer explanation of how potential biases were mitigated in the methodology.

2. Formulation of the Conclusion
The conclusion, both in the abstract and the main text, should provide a direct and concise answer to the study's primary objective. While the results section presents the data, the conclusion must synthesize these findings to explicitly state how they address the aim of analyzing mortality patterns using the two methodologies. A summary of the distinct patterns identified for each methodology should be clearly articulated in the concluding remarks.

3. Application of the RECORD Checklist
Given that this study utilizes large, routinely collected health databases, the application of the RECORD checklist (Benchmark et al., 2015) is highly recommended. This checklist is an extension of STROBE specifically for such datasets.

* Key items to address include providing a flow diagram illustrating the patient selection and data linkage process (which could be included as a supplement).
* Furthermore, a detailed description of the data cleaning and preparation procedures is essential. This should cover the handling of missing data, inconsistencies, and coding errors, including the percentage of missing data and the methods used for its treatment (e.g., imputation, exclusion).

4. Application of the RECORD-PE Checklist

Finally, due to the perinatal focus of the research, I strongly suggest applying the RECORD-PE checklist (Perinatal Extension, Hermanussen et al., 2021). This extension is tailored for observational studies involving maternal, fetal, and newborn data.

* For instance, the methodology should explicitly describe how specific perinatal data challenges were handled. This includes the treatment of live births without a linked death record, death records without a corresponding birth date, and the linkage accuracy between maternal and neonatal records.
