# Supplementary material for: A two-tiered latent class and spatial analytical approach to identify clusters of neonatal mortality among very low birth weight infants: A population-based cohort study
Source: PLoS One. 2026 Feb 24;21(2):e0343004. doi: 10.1371/journal.pone.0343004 (PMC12931791; doi:10.1371/journal.pone.0343004)
Supplement: Attachment — Submitted filename: PONE-D-25-47659_Response_23jan26.docx [file pone.0343004.s004.docx]

**Response to Reviewers**

**Manuscript title:** A two-tiered class and spatial analytical approach to identify clusters of neonatal mortality among very low birth weight infants: a population-based cohort study

**Manuscript ID:** PONE-D-25-47659
**Corresponding author:** Daniela Testoni Costa-Nobre

Dear Dr. Gurgel,
Dear Academic Editor and Reviewers,

We would like to thank you and the reviewers for the careful evaluation of our manuscript contributing to the overall quality of our work. We are grateful for the positive assessment of the scientific merit, methodological rigor, and relevance of the study.

Below, we provide a detailed, point-by-point response to all comments raised by the Academic Editor and the reviewers. All changes have been incorporated into the revised manuscript and are highlighted in the tracked-changes version. Page and line numbers refer to the revised manuscript.

**Responses to Journal and Editorial Requirements**

1. Please ensure that your manuscript meets PLOS ONE's style requirements, including those for file naming. The PLOS ONE style templates can be found at

https://journals.plos.org/plosone/s/file?id=wjVg/PLOSOne_formatting_sample_main_body.pdf and <https://journals.plos.org/plosone/s/file?id=ba62/PLOSOne_formatting_sample_title_authors_affiliations.pdf>

**Response:**
We have revised the manuscript to fully comply with PLOS ONE formatting and style guidelines. File naming, structure, headings, tables, and figures were checked and adjusted according to the official PLOS ONE templates.

2. We note that the grant information you provided in the ‘Funding Information’ and ‘Financial Disclosure’ sections do not match.

When you resubmit, please ensure that you provide the correct grant numbers for the awards you received for your study in the ‘Funding Information’ section.

Discrepancies were identified between the Funding Information, Financial Disclosure, and text within the manuscript.

**Response:**
We appreciate the editor’s careful review of the funding information. We have taken the following actions:

- All funding-related text has been removed from the manuscript body and Acknowledgments section, in accordance with PLOS ONE policy.
- The Funding Statement has been corrected and standardized.
- The Financial Disclosure has been clarified to explicitly state the role of the funders.

3. Thank you for stating the following financial disclosure:

[The author(s) received no specific funding for this work.].

At this time, please address the following queries:

a) Please clarify the sources of funding (financial or material support) for your study. List the grants or organizations that supported your study, including funding received from your institution.

b) State what role the funders took in the study. If the funders had no role in your study, please state: “The funders had no role in study design, data collection and analysis, decision to publish, or preparation of the manuscript.”

c) If any authors received a salary from any of your funders, please state which authors and which funders.

d) If you did not receive any funding for this study, please state: “The authors received no specific funding for this work.”

Please include your amended statements within your cover letter; we will change the online submission form on your behalf.

We have taken the following actions:

- All funding-related text has been removed from the manuscript body and Acknowledgments section, in accordance with PLOS ONE policy.
- The Funding Statement has been corrected and standardized.
- The Financial Disclosure has been clarified to explicitly state the role of the funders.

Funding was provided by Fundação de Amparo à Pesquisa do Estado de São Paulo (FAPESP), Project #2017/03748-7. The funding agency did not interfere with or participate in the study design, data analysis, interpretation of the results, or the writing and revision of the manuscript. The authors received no other specific funding for this work. Use of the data was possible due to agreements between Fundação SEADE and Universidade Federal de São Paulo (#23089.004297/2008-11; #23089.000057/2014-95; #23089.120254/2019-34). The authors have declared that no competing interests exist.

4. Thank you for stating the following in your manuscript:

[Fundação de Amparo à Pesquisa de São Paulo - FAPESP, Project # 2017/03748-7. The funding agency did not interfere or participate in the design of the study, data analysis, interpretation of the results, writing or revising of the manuscript.]

We note that you have provided funding information that is not currently declared in your Funding Statement. However, funding information should not appear in the Acknowledgments section or other areas of your manuscript. We will only publish funding information present in the Funding Statement section of the online submission form.

Please remove any funding-related text from the manuscript and let us know how you would like to update your Funding Statement. Currently, your Funding Statement reads as follows:

[The author(s) received no specific funding for this work.]

Please include your amended statements within your cover letter; we will change the online submission form on your behalf.

We have adjusted as mentioned above:

Funding was provided by Fundação de Amparo à Pesquisa do Estado de São Paulo (FAPESP), Project #2017/03748-7. The funding agency did not interfere with or participate in the study design, data analysis, interpretation of the results, or the writing and revision of the manuscript. The authors received no other specific funding for this work. Use of the data was possible due to agreements between Fundação SEADE and Universidade Federal de São Paulo (#23089.004297/2008-11; #23089.000057/2014-95; #23089.120254/2019-34). The authors have declared that no competing interests exist.

5. Thank you for stating the following in your Competing Interests section:

[No authors have competing interests].

Please complete your Competing Interests on the online submission form to state any Competing Interests. If you have no competing interests, please state "The authors have declared that no competing interests exist.", as detailed online in our guide for authors at http://journals.plos.org/plosone/s/submit-now

This information should be included in your cover letter; we will change the online submission form on your behalf.

We have completed the **Competing Interests** section in the online submission form as requested and included the information in the cover letter. The statement has been updated to read as follows:

Funding was provided by Fundação de Amparo à Pesquisa do Estado de São Paulo (FAPESP), Project #2017/03748-7. The funding agency did not interfere with or participate in the study design, data analysis, interpretation of the results, or the writing and revision of the manuscript. The authors received no other specific funding for this work. Use of the data was possible due to agreements between Fundação SEADE and Universidade Federal de São Paulo (#23089.004297/2008-11; #23089.000057/2014-95; #23089.120254/2019-34). The authors have declared that no competing interests exist.

If the reviewer comments include a recommendation to cite specific previously published works, please review and evaluate these publications to determine whether they are relevant and should be cited. There is no requirement to cite these works unless the editor has indicated otherwise.

We reviewed the reviewer comments: no specific previously published works were recommended for citation.

Please review your reference list to ensure that it is complete and correct. If you have cited papers that have been retracted, please include the rationale for doing so in the manuscript text, or remove these references and replace them with relevant current references. Any changes to the reference list should be mentioned in the rebuttal letter that accompanies your revised manuscript. If you need to cite a retracted article, indicate the article’s retracted status in the References list and also include a citation and full reference for the retraction notice.

We carefully reviewed the reference list to ensure completeness and accuracy. No retracted articles were cited in the manuscript. In response to the RECORD checklist requirements, two additional references were included to support methodological and reporting aspects addressed in the revision. The inclusions are below:

Ref 10

Waldvogel BC, Morais LCC, Perdigão ML, Teixeira MLP, Freitas RMV, Aranha VJ. Experiência da Fundação Seade com a aplicação da metodologia de vinculação determinística de bases de dados. Ensaio & Conjuntura. 2019;1:1-25

Ref 12

Areco KN, Konstantyner T, Bandiera-Paiva P, Balda RCX, Costa-Nobre DT, Sanudo A, et al. Operational Challenges in the Use of Structured Secondary Data for Health Research. Front Public Health. 2021 Jun 15;9:642163. doi: 10.3389/fpubh.2021.642163.

**Reviewer #1**

The submitted study aims to identify and analyze patterns of neonatal deaths among very low birth weight newborns in the state of São Paulo, Brazil. It is a robust investigation that examined more than 100,000 live births and over 40,000 neonatal deaths over a 16-year period. Relevant patterns were identified using a concise methodology and a rigorous statistical analysis, employing two statistical approaches that are appropriate to the research topic and objectives. As expected within the Brazilian context, regional disparities and persistent problems were observed, which require attention and targeted efforts toward addressing preventable factors, as highlighted by the authors. This is a topic of utmost importance that should be brought to academic discussion.

**Response:**

We thank Reviewer #1 for the positive and thoughtful evaluation of our study. We appreciate the recognition of its methodological rigor, analytical approach, and public health relevance, particularly regarding neonatal mortality patterns and regional disparities.

**Reviewer #2**

**1. Adherence to the STROBE Statement.** To ensure comprehensive reporting and identification of best practices, I recommend the authors explicitly confirm that all items of the STROBE checklist have been addressed.

Item 4 (Title and Abstract): The manuscript's title should clearly state the study design. While the use of Latent Class Analysis implies an observational design, the specific type (which I understand to be a retrospective cohort study) should be explicitly stated in the title, or if not possible, the abstract. This is a key requirement for immediate reader comprehension.

Other Items: Certain other STROBE criteria appear to be only partially met. These include a more detailed description of the sampling method and a clearer explanation of how potential biases were mitigated in the methodology.

**Response:**
To clearly identify the study design, and to include the suggestion of reviewer #3, we have revised the manuscript title from *“Latent class and spatial analyses in a tiered approach for identifying cluster areas of early or late neonatal mortalities”* to “A two-tiered class and spatial analytical approach to identify clusters of neonatal mortality among very low birth weight infants: a population-based cohort study.”

In addition, we explicitly stated the study design in the abstract (Page 2, Line 58) by adding the terms *retrospective* and *cohort* (“This retrospective population-based cohort study…”), and in the Methods section (Page 4, Line 120) (“This was a retrospective population-based cohort study using data from deterministic linkage…”).

We have reviewed the manuscript using the STROBE checklist to ensure comprehensive reporting of all recommended items. A completed STROBE checklist is now provided as supplementary material.

**2. Formulation of the Conclusion**

The conclusion, both in the abstract and the main text, should provide a direct and concise answer to the study's primary objective. While the results section presents the data, the conclusion must synthesize these findings to explicitly state how they address the aim of analyzing mortality patterns using the two methodologies. A summary of the distinct patterns identified for each methodology should be clearly articulated in the concluding remarks.

**Response:**
We thank the reviewer for this valuable suggestion and agree that the conclusion should more directly and concisely address the study’s primary objective. In response, we rewrote the conclusions in both the abstract and the main text to explicitly synthesize the findings and clarify how the combined methodologies address the aim of analyzing neonatal mortality patterns.

In the abstract (Page 2, Line 65), the conclusion was revised to state:

*“The combined use of LCA and spatial analysis identified distinct patterns of neonatal mortality. LCA differentiated clinically meaningful profiles with specific timing of death, while spatial analysis revealed municipal-level clustering and overlap of these patterns. These findings showed how neonatal mortality is shaped by both diagnostic profiles and territorial context, providing actionable evidence to guide targeted improvements in perinatal and neonatal care to reduce preventable deaths among VLBW infants.”*

In the main text (Page 16, Line 424), the conclusion was rewritten as follows:

*“In conclusion, by applying a two-tiered approach, this study highlights how the combination of methodological rigor and epidemiological insight can uncover diagnostic and geographic patterns in neonatal mortality. Latent class analysis identified distinct and clinically meaningful diagnostic patterns, differentiating deaths predominantly related to intrapartum events from those associated with infections, respiratory conditions, and congenital malformations, with clear differences in timing of death. Spatial analysis further demonstrated that these classes were unevenly distributed across municipalities, revealing geographic clustering and overlap of specific mortality patterns that reflect heterogeneity in health system performance.”*

We believe these revisions directly respond to the reviewer’s concern by clearly summarizing the distinct contributions of each methodology and explicitly linking the findings to the study’s primary objective.

**3. Application of the RECORD Checklist**

Given that this study utilizes large, routinely collected health databases, the application of the RECORD checklist (Benchmark et al., 2015) is highly recommended. This checklist is an extension of STROBE specifically for such datasets.
* Key items to address include providing a flow diagram illustrating the patient selection and data linkage process (which could be included as a supplement).
* Furthermore, a detailed description of the data cleaning and preparation procedures is essential. This should cover the handling of missing data, inconsistencies, and coding errors, including the percentage of missing data and the methods used for its treatment (e.g., imputation, exclusion).

**Response:**
We have addressed this recommendation in full:

The RECORD checklist has been completed and included as Supplementary Material.

We change the manuscript to better adhere to the RECORD checklist

*RECORD 6.2: Any validation studies of the codes or algorithms used to select the population should be referenced. If validation was conducted for this study and not published elsewhere, detailed methods and results should be provided.

Data preparation and validation were conducted through a complex and time-consuming process that has been previously published. For clarity, we have now added the sentence below along with the corresponding reference. (Ref 12)

We added in Page 4 Line 126: “Procedures to ensure data adequacy, including data preparation and data validation prior to data use, were undertaken and have been previously published.”

Areco KN, Konstantyner T, Bandiera-Paiva P, Balda RCX, Costa-Nobre DT, Sanudo A, et al. Operational Challenges in the Use of Structured Secondary Data for Health Research. Front Public Health. 2021 Jun 15;9:642163. doi: 10.3389/fpubh.2021.642163.

* RECORD 6.3: If the study involved linkage of databases, consider use of a flow diagram or other graphical display to demonstrate the data linkage process, including the number of individuals with linked data at each stage.

The deterministic data linkage procedures used in this study have been previously described in detail. Accordingly, we referenced the original methodological source (Reference #10), which provides a comprehensive description of the linkage process.

Waldvogel BC, Morais LCC, Perdigão ML, Teixeira MLP, Freitas RMV, Aranha VJ. Experiência da Fundação Seade com a aplicação da metodologia de vinculação determinística de bases de dados. Ensaio & Conjuntura. 2019;1:1-25

RECORD 12.1: Authors should describe the extent to which the investigators had access to the database population used to create the study population.

The investigators had full access to the de-identified population-based database used to create the study population. Data were provided by the State Data Analysis System Foundation (Fundação SEADE) and included all eligible live births and neonatal deaths registered in São Paulo State during the study period. The dataset contained individual-level records with all variables required for deterministic linkage, latent class analysis, and spatial analysis, without restrictions on data use for approved research purposes. All analyses were conducted by the study investigators using these complete datasets.

We now added (Page 4 Line 125) the sentence: “We had full access to the de-identified population-based database used to create the study population.”

RECORD 12.2: Authors should provide information on the data cleaning methods used in the study.

Data preparation and validation were conducted through a complex and time-consuming process that has been previously published as explained above (Ref 12).

RECORD 19.1: Discuss the implications of using data that were not created or collected to answer the specific research question(s). Include discussion of misclassification bias, unmeasured confounding, missing data, and changing eligibility over time, as they pertain to the study being reported.

These limitations are discussed in Pages 15 and 16 and we added a new sentence in the discussion for clarity (Line 419): “As this study relied on routinely collected secondary data not originally designed to address the specific research question, potential misclassification, unmeasured confounding, and temporal changes in care practices should be considered when interpreting the findings.”

**4. Application of the RECORD-PE Checklist**

Finally, due to the perinatal focus of the research, I strongly suggest applying the RECORD-PE checklist (Perinatal Extension, Hermanussen et al., 2021). This extension is tailored for observational studies involving maternal, fetal, and newborn data.

**Response:**
We thank the reviewer for this suggestion. We carefully searched for a perinatal extension checklist specifically designed for RECORD and did not identify a general *Perinatal Extension* applicable to observational studies of neonatal outcomes based on routinely collected data. The only RECORD-PE identified was RECORD-PE that extends for Pharmacoepidemiology Extension.

<https://www.record-statement.org/checklist-pe.php>

As our study does not evaluate pharmacological exposures, medication use, or treatment windows, several key items of RECORD-PE (e.g., drug exposure definition, exposure risk windows, and medication-specific confounding) are not applicable. Therefore, we maintained adherence to the STROBE and RECORD guidelines, which are more appropriate for the population-based design and data sources used in this study and adequately cover the relevant reporting domains.

**Reviewer #3**

We thank Reviewer #3 for the insightful comments aimed at strengthening the clarity, and coherence of the manuscript.

**Title**

 I would suggest adjusting the phrasing as: “A two-tiered latent class and spatial analytical approach to identify clusters of early and late neonatal mortality among very low birth weight infants”

**Response:** We appreciate the reviewer´s suggestion and have implemented the proposed changes. In line with Reviewer #1´s recommendation, we explicitly included the study design. Accordingly, the title was *“Latent class and spatial analyses in a tiered approach for identifying cluster areas of early or late neonatal mortalities”* to “A two-tiered class and spatial analytical approach to identify clusters of neonatal mortality among very low birth weight infants: a population-based cohort study.”

**Abstract**

Objective:
Line 27: …patterns of neonatal deaths among very low birth weight… maintain consistency between title (early/late mortality) and the abstract (general neonatal mortality)
We thank the reviewer for the observation. We would like to clarify that the primary objective of the study was to identify and analyze overall patterns of neonatal mortality among very low birth weight infants using latent class and spatial analyses, without a priori stratification by early or late neonatal death.

The distinction between early and late neonatal mortality emerged as a finding of the latent class analysis, reflecting differences in diagnostic profiles and timing of death across the identified classes. To ensure consistency with the study objective, we revised the manuscript title and removed references to early and late neonatal mortality.

Introduction
Line 58 spatial-temporal- better to replace with: spatio-temporal
Line 152: 34% were <28 weeks... refine as: “… 34% were born before 28 weeks of gestation.” To clarify the reference to gestational age.

**Response:**
All suggested edits have been implemented, including:

- Spatial-temporal changed to spatio-temporal (now line 79)
- <28 weeks... changed as: “… 34% were born before 28 weeks of gestation

**Methods**

The authors may need more detailed justification why the four diagnostic groups were selected (infection, intrapartum event, malformation, respiratory) as they are broad and include overlapping ICD codes.

**Response:**
We thank the reviewer for this comment. The four diagnostic groups (infection, intrapartum events, malformations, and respiratory conditions) were selected a priori because they represent the major and programmatically distinct causes of neonatal mortality, particularly among very low birth weight infants, and are consistently reported in the literature as reflecting different etiological pathways, timing of death, and points of intervention along the continuum of perinatal and neonatal care.

Although these groups are broad and may include overlapping ICD-10 codes, this was intentional to reflect the multifactorial nature of neonatal deaths and the frequent coexistence of multiple diagnoses on death certificates. Rather than enforcing mutually exclusive categories at the coding level, this complexity was addressed analytically using latent class analysis, which identifies diagnostic patterns based on the co-occurrence of conditions.

We have clarified this rationale in the Methods section adding the following sentence (line 129): “Diagnostic groups were defined a priori to represent major etiological pathways of neonatal mortality and were intentionally broad to capture overlapping diagnoses, allowing latent class analysis to probabilistically identify clinically meaningful patterns of co-occurring conditions.”

**Results**
The paper would benefit from clearly identifying which classes are the main drivers of overall spatial disparities in neonatal mortality across the state.

The results are reported by class, but the implications for how these patterns translate into health system gaps remain somewhat implicit. Strengthening the link between (1) each class’s spatial clustering, (2) the likely underlying service-delivery challenges, and (3) the most relevant targeted interventions would enhance the translational value and clarity of the findings.

**Response:**
We thank the reviewer for the suggestion. In response, we added a paragraph in the Discussion section (Line 336) to more clearly identify which latent classes are the main drivers of spatial disparities in neonatal mortality. We now highlight that classes dominated by intrapartum-related conditions and infections exhibited the most pronounced and geographically concentrated high–high clusters, suggesting a stronger contribution to overall spatial inequality.

We further strengthened the interpretation by explicitly linking each class’s spatial clustering to likely service-delivery gaps and corresponding targeted interventions, including improvements in intrapartum care, referral pathways, NICU capacity, and infection prevention and control.

The paragraph inserted in line 336 is as follows: *“The spatial analysis indicated that latent classes dominated by intrapartum-related conditions and infections were the main contributors to geographic disparities in neonatal mortality, as they showed the most consistent and concentrated high–high clusters across municipalities. These patterns likely reflect deficiencies in the quality of antenatal and intrapartum care, delays in referral, and limitations in neonatal intensive care and infection prevention practices. In contrast, classes characterized predominantly by congenital malformations and respiratory conditions exhibited more diffuse or overlapping spatial patterns, suggesting a lesser contribution to overall spatial inequality. By linking class-specific spatial clustering to plausible health system gaps, these findings provide actionable guidance for targeted interventions aimed at reducing preventable neonatal deaths.”*

**Discussion**

The Discussion could better detail how referral bias may over- or under-estimate clustering. Linking between each latent class and actionable strategies could be articulated more clearly. Providing class-specific examples (e.g., infection prevention bundles vs. improvements in delivery-room care) would strengthen policy relevance.

**Response:**
We agree with the reviewer that referral can affect cluster areas. The Discussion was expanded to explicitly address referral bias, noting that analyses based on municipality of death may overestimate clustering in referral centers for late deaths (particularly infection- and respiratory-dominant classes) and underestimate clustering for early intrapartum-related deaths occurring before transfer.

In addition, we strengthened the link between latent class–specific spatial patterns and actionable strategies, as detailed in our previous response, by explicitly connecting intrapartum-dominant classes to antennal and intrapartum care improvements, and infection-dominant classes to infection prevention bundles, surveillance, and NICU-based interventions.

We included the following paragraph in the discussion (Line 345): “The spatial analysis may be influenced by referral bias, as clustering based on municipality of death can overestimate rates in municipalities hosting referral centers, particularly for infection- and respiratory-dominant classes associated with later neonatal deaths. Conversely, early intrapartum-related deaths may be underestimated in these areas, as deaths often occur before inter-municipality transfer. Importantly, class-specific spatial clustering offers actionable insights: intrapartum-dominant clusters point to the need for strengthening antenatal and intrapartum care, whereas infection-dominant clusters highlight priorities for infection prevention bundles, antimicrobial stewardship, and enhanced surveillance within neonatal intensive care units.”

**Conclusion**
Given the paper’s title, the conclusion should more explicitly highlight what the study reveals about early vs late neonatal deaths across latent classes, based on the survival curves.

We thank the reviewer for this comment. In response to Reviewer #2, the conclusion has already been revised to more explicitly highlight the study’s findings regarding early versus late neonatal deaths across latent classes, as informed by the survival curve analyses.

In the abstract (line 65 page 2), the conclusion was revised to state:

*“The combined use of LCA and spatial analysis identified distinct patterns of neonatal mortality. LCA differentiated clinically meaningful profiles with specific timing of death, while spatial analysis revealed municipal-level clustering and overlap of these patterns. These findings showed how neonatal mortality is shaped by both diagnostic profiles and territorial context, providing actionable evidence to guide targeted improvements in perinatal and neonatal care to reduce preventable deaths among VLBW infants.”*

In the main text (line 424, page 16), the conclusion was rewritten as follows:

*“In conclusion, by applying a two-tiered approach, this study highlights how the combination of methodological rigor and epidemiological insight can uncover diagnostic and geographic patterns in neonatal mortality. Latent class analysis identified distinct and clinically meaningful diagnostic patterns, differentiating deaths predominantly related to intrapartum events from those associated with infections, respiratory conditions, and congenital malformations, with clear differences in timing of death. Spatial analysis further demonstrated that these classes were unevenly distributed across municipalities, revealing geographic clustering and overlap of specific mortality patterns that reflect heterogeneity in health system performance.”*

Once again, we sincerely thank the Academic Editor and reviewers for their valuable feedback. We believe that the revisions have significantly strengthened the manuscript in terms of transparency, reporting quality, and policy relevance. We hope that the revised version now fully meets the publication criteria of PLOS ONE.

Kind regards,

**Daniela Testoni Costa-Nobre**
On behalf of all authors
